# 4D VMAT planning and verification technique for dynamic tracking using a direct aperture deformation (DAD) method

**DOI:** 10.1002/acm2.12053

**Published:** 2017-03-06

**Authors:** Yongqian Zhang, Yong Yang, Weihua Fu, Xiang Li, Tianfang Li, Dwight E. Heron, M. Saiful. Huq

**Affiliations:** ^1^ Department of Radiation Oncology University of Pittsburgh Cancer Institute Pittsburgh PA 15232 USA; ^2^ Department of Radiation Oncology Stanford University Stanford CA 94305 USA; ^3^ Department of Radiation Oncology Memorial Sloan‐Kettering Cancer Center New York NY 10065 USA

**Keywords:** 4D VMAT, direct aperture deformation (DAD), IMRT verification, stereotactic ablative body radiotherapy (SABR)

## Abstract

We developed a four‐dimensional volumetric modulated arc therapy (4D VMAT) planning technique for moving targets using a direct aperture deformation (DAD) method and investigated its feasibility for clinical use. A 3D VMAT plan was generated on a reference phase of a 4D CT dataset. The plan was composed of a set of control points including the beam angle, MLC apertures and weights. To generate the 4D VMAT plan, these control points were assigned to the closest respiratory phases using the temporal information of the gantry angle and respiratory curve. Then, a DAD algorithm was used to deform the beam apertures at each control point to the corresponding phase to compensate for the tumor motion and shape changes. Plans for a phantom and five lung cases were included in this study to evaluate the proposed technique. Dosimetric comparisons were performed between 4D and 3D VMAT plans. Plan verification was implemented by delivering the 4D VMAT plans on a moving QUASAR™ phantom driven with patient‐specific respiratory curves. The phantom study showed that the 4D VMAT plan generated with the DAD method was comparable to the ideal 3D VMAT plan. DVH comparisons indicated that the planning target volume (PTV) coverages and minimum doses were nearly invariant, and no significant difference in lung dosimetry was observed. Patient studies revealed that the GTV coverage was nearly the same; although the PTV coverage dropped from 98.8% to 94.7%, and the mean dose decreased from 64.3 to 63.8 Gy on average. For the verification measurements, the average gamma index pass rate was 98.6% and 96.5% for phantom 3D and 4D VMAT plans with 3%/3 mm criteria. For patient plans, the average gamma pass rate was 96.5% (range 94.5–98.5%) and 95.2% (range 94.1–96.1%) for 3D and 4D VMAT plans. The proposed 4D VMAT planning technique using the DAD method is feasible to incorporate the intra‐fraction organ motion and shape change into a 4D VMAT planning. It has great potential to provide high plan quality and delivery efficiency for moving targets.

**Abbreviations**
4D VMAT: four‐dimensional volumetric modulated arc therapy
6X‐FFF: 6 MV flattening filter free beam
AAPM: American Association of Physicists in Medicine
DAD: direct aperture deformation
GTV: gross tumor volume
MLC: multi‐leaf collimator
OAR: organ at risk
PTV: planning tumor volume
SABR: stereotactic ablative body radiotherapy
TPS: treatment planning system
VMAT: volumetric modulated arc therapy

## 1 Introduction

Volumetric Modulated Arc Therapy (VMAT) is delivered through synchronized variation in the gantry angle, dose rate, and multi‐leaf collimator (MLC) leaf positions.^1^ Studies have shown that VMAT can provide high delivery efficiency without compromising plan quality compared to static beam IMRT.^2^, ^3^, ^4^, ^5^ Verbakel et al.^6^ have shown that for patients with Stage I lung cancer, the VMAT stereotactic ablative body radiotherapy (SABR) technique achieves better target dose conformity than a conventional 10‐field non‐coplanar IMRT plan. However, tumor motion due to respiration during radiation therapy for cancer radiotherapy is a significant problem. The compilation of data in the American Association of Physicists in Medicine (AAPM) Task Group Report 76^7^ revealed that out of 22 lung tumor patients, 12 patients had tumor motion from 3 to 22 mm (mean 8 ± 4 mm) in the Superior–Inferior direction. In such a situation, the delivered dose distribution could be different from the original planned dose distribution if the intra‐fraction tumor and organs‐at‐risk motions were not taken into account properly.^7,8,9^

Several methods have been proposed to manage the intra‐fraction tumor motion, including margin expansion,^10^ gating techniques^11^, ^12^, ^13^, ^14^ and tracking techniques.^15^, ^16^, ^17^ Important considerations for SABR treatment include minimizing the volume of the normal tissues outside the tumor receiving high doses per‐fraction and achieving acceptable dose inhomogeneity inside the tumor. Therefore, the common use of large treatment margins in lung cancer is in conflict with SABR's requirement of minimal treatment field sizes.^10^ Gating techniques reduce the volume of healthy tissue exposed to high doses of radiation.^11^, ^12^, ^13^, ^14^ However, gating techniques have limited beam output, therefore, gating techniques increase the treatment delivery time especially for SABR treatments. Rigid tracking techniques can be used to compensate for tumor motion but cannot deal with deformable motion effects.^15^, ^16^, ^17^

Four‐dimensional volumetric modulated arc therapy (4D VMAT) is a treatment strategy for lung cancers that aims to exploit relative target and tissue motion to improve target coverage and organ at risk (OAR) sparing.^18^, ^19^, ^20^ With the development of sophisticated imaging techniques that provide information on tumor motion and deformation, such as 4D‐CT^21^, ^22^, ^23^and 4D‐CBCT,^24^, ^25^, ^26^ the 4D plan optimization strategy presents a logical solution to account for the intra‐fractional organ motion. An inverse planning framework for 4D VMAT was proposed by Ma^18^ to provide tempo‐spatially optimized VMAT plans. The cumulative dose distribution was optimized by iteratively adjusting the aperture shape and weight of each beam through the minimization of the planning objective function. The proposed 4D VMAT planning formulism provided useful insight on how the “time” dimension could be exploited in rotational arc therapy to maximally compensate for the intra‐fraction organ motion. Chin^19^, ^20^ investigated a novel algorithm for true 4D‐VMAT planning by incorporating the 4D volumetric target and OAR motions directly into the optimization process. During optimization, phase correlated beam samples were progressively added throughout the full range of gantry rotation. The resulting treatment plans had respiratory phase‐optimized apertures whose deliveries were synchronized to the patient's respiratory cycle. The 4D VMAT system has the potential to improve radiation therapy of periodically moving tumors over 3D VMAT, gating or tracking methods. However, the complex dose calculation and optimization may prolong the treatment planning time and cannot be implemented on commercial treatment planning systems.

In this work, we propose a 4D VMAT planning technique by applying a direct aperture deformation algorithm to a 3D VMAT plan. This method accounts for both the rigid and non‐rigid respiration‐induced target motion and is simple and feasible for clinical setup.

## 2 Methods

**Figure 1:**
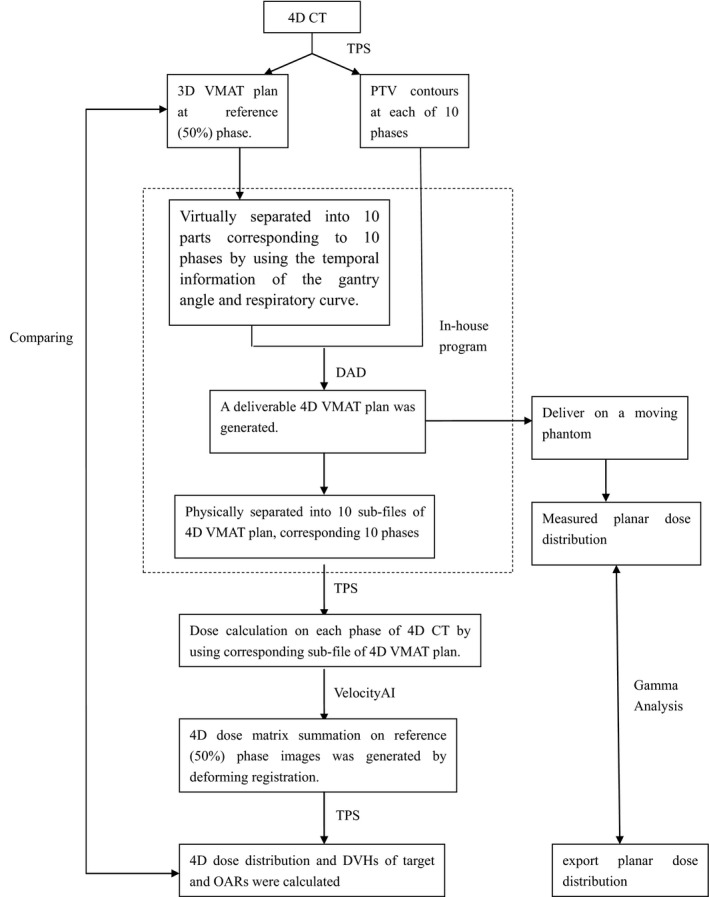
The scheme of this study, including 4D treatment planning, dosimetric comparison and plan verification.

Plans for a QUASAR™ phantom with a tumor insert and for five patients who received lung SABR treatments were included in this study. Figure 1 shows the scheme of this study from 4D CT to 4D VMAT plan verification. First, a 3D VMAT plan was optimized based on patient's anatomy on the reference (50%) phase of a 4D CT dataset using Eclipse treatment planning system. The 3D VMAT plans consisted of a sequence of control points each defining the gantry angle, dose weight, and MLC aperture, the gantry speed for each control point was also calculated as can be seen from the beam properties for each control point in Eclipse. Second, the gantry angle for each control point generated from the 3D VMAT plans could be used to link the plan time points and the tumor motion, which is illustrated in the next paragraph. Once the 4D VMAT plan and the tumor motion was synchronized, the DAD method was used to modify the MLC leaf positions at each control point of the plan to synchronize the VMAT delivery with the respiratory motion. Third, the quality of the resultant 4D VMAT plan was investigated by comparing its isodose distribution and DVHs with the 3D VMAT plan. Fourth, plan verification was implemented by delivering the 4D VMAT plans on a moving QUASAR™ phantom driven with patient‐specific respiratory curves.

The gantry angle and gantry speed information could be used to synchronize the plan time points with the phase of breathing motion. Since the only difference between the 3D and the 4D VMAT plans was the MLC apertures, and the dose rate for each control point was less than the maximum value, therefore, the 4D VMAT plans could be delivered with the same gantry angle and gantry speed for each control point once the MLC leaf travel speed be constrained to a value less than the physical maximum speed. (a) During the 3D VMAT optimization, preserving the maximum speed of leaf motion to below the speed of *v*_max_ had to be compromised such that the leaf velocity in the target‐reference frame could be constrained to *v*_max_. The MLC leaf travel speed was set to 1.5 cm/s for 3D VMAT planning optimization in this study; other planning parameters were gantry speed 0.5 to 4.8 degrees/s, and dose rate 0 to 1400 MU/min, and the physical maximum leaf travel speed 2.5 cm/s (b) once the 4D VMAT plan was generated based on the DAD method, the speed of a MLC leaf at position X as a function of gantry angle g, V(g) = dX/dg, could be related with gantry speed dg/dt and MLC physical leaf speed as follows

$$V\left(g\right)=\frac{dX}{dg}=\frac{dX}{dt}\cdot \frac{dt}{dg}$$

Where $\frac{dX}{dt}$ denotes the leaf travel speed and $\frac{dt}{dg}$ denotes the reciprocal of gantry speed. The MLC leaf speed should be less than 2.5 $\frac{dt}{dg}$ at each control point. (c) We compared the gantry angles recorded at each control point within the trajectory log files with the 3D and the 4D VMAT plans. Once the 4D VMAT plan could not be delivered with the planned gantry speed due to limited leaf travel speed, the MLC leaf position at that control point had to be modified such that the 4D VMAT plan deliveries could be synchronized with the breathing motion.

### 2.A Plan preparation

4D CT images were acquired on a GE Discovery PET/CT scanner. Audio coaching was used to improve the reproducibility and stability of the breathing motion. For the phantom study, the QUASAR™ phantom was driven by a periodic sinusoidal curve with the motion amplitude of 1.0 cm and the motion cycle of 5 s. The 4D CT images were imported into the Varian Eclipse treatment planning system (TPS) for contouring and treatment planning. The gross tumor volumes (GTVs) were delineated on each of the ten respiratory phases of the 4D CT. The planning target volumes were defined as the GTVs plus a 5 mm isotropic margin. The amplitude of tumor motion was determined by measuring the peak‐to‐peak tumor position from different phases of the breathing cycle for each patient. The target volumes and motion amplitudes are listed in Table 1. For the 5 patients in this study, the tumor motion was greater than 5 mm. The prescription dose to the PTV was 60 Gy to be delivered in three fractions with a 6 MV Flattening Filter Free (6X‐FFF) X‐ray beam from a TrueBeam™STx linear accelerator. The prescribed isodose line was individually selected for each plan such that at least 95% of the PTV was covered by the prescription dose. In our study, the 50% respiratory phase of the 4D CT image sets (corresponding to end exhalation) was selected as the reference image for 3D VMAT planning and dose verification.

**Table 1:** Target volumes and motion amplitudes in studied cases

Case no.	GTV volume (cm^3^)	PTV volume (cm^3^)	Motion amplitude (mm)
1	2.2	9.9	16
2	0.8	5.5	7.0
3	0.8	6.0	6.5
4	0.9	7.2	6.0
5	16.1	40.9	10

### 2.B 4D VMAT plan generation algorithm

A DAD method is used to modify the MLC leaf positions at each control point to synchronize the VMAT plan delivery with the respiratory motion. The target translation and shape deformation are taken into account in the modification while the total monitor unit (MU) for each beam and the MU fraction and gantry angle for each control point are kept unchanged as those in the original plan. Once the correlation between the Gantry angle and the target position from the 4D CT scan is established using the temporal information of the gantry angle and respiratory curve, the projected outlines for both reference phase (50% phase) and the target phase (Nth phase) in the BEV at the gantry angle of the corresponding control point are generated using an in‐house program. To modify the MLC aperture from the reference phase to the Nth phase, the first step is to calculate the shift in the X‐direction (corresponding to the right–left direction of the patient) in terms of geometric center of the projected outlines (the collimator is set to 90° for all the plans). This shift is accounted for by moving the open subfields right or left by an integral number (*k*) of MLC leaves. The *k* is determined by the quotient of the shift in the X‐direction and the width of MLC leaf. Therefor the (*i* + *k*)th leaf pair in the new plan is corresponding to the *i*th leaf pair in the original plan. The (*i*+*k*)th leaf pair positions in the new beam are calculated by

(1) $$\begin{array}{c} A_{i+k}^{N}=\;\left(A_{i}^{O}-Y_{i}^{O}\right)\times Scale_{i}+Y_{i+k}^{N}\;\text{and}\\ B_{i+k}^{N}=\left(B_{i}^{O}-Y_{i}^{O}\right)\times Scale_{i}+Y_{i+k}^{N} \end{array}$$

Where *A*_*i*_ and *B*_*i*_ are the position of the leading and trailing leaves of the *i*th leaf pair. The superscript “O” stands for the target and leaf sequence in the original plan. The superscript “N” stands for the target and new leaf sequence for the *N*th Phase. *Y*_*i*_ is the geometric center of the projected outline in the Y‐direction under the *i*th leaf pair and can be obtained by

(2) $$Y_{i}^{O}=\frac{Y_{Si}^{O}+Y_{Ii}^{O}}{2}\;\;\text{and}\;\;Y_{i+k}^{N}=\frac{Y_{S(i+k)}^{N}+Y_{I(i+k)}^{N}}{2}$$

While $Y_{Si}^{O}$ and $Y_{Ii}^{O}$ are the superior and inferior boundaries of the outline projection in the Y‐direction under the *i*th leaf pair for the original plan, $Y_{S(i+k)}^{N}$ and $Y_{I(i+k)}^{N}$ are the superior and inferior boundaries of the outline projection in the Y‐direction under the (*i + k*)th leaf pair for the Nth phase. *Scale*_*i*_ is calculated by

(3) $$Scale_{i}=\frac{Y_{S(i+k)}^{N}-Y_{I(i+k)}^{N}}{Y_{Si}^{O}-Y_{Ii}^{O}}$$

**Figure 2:**
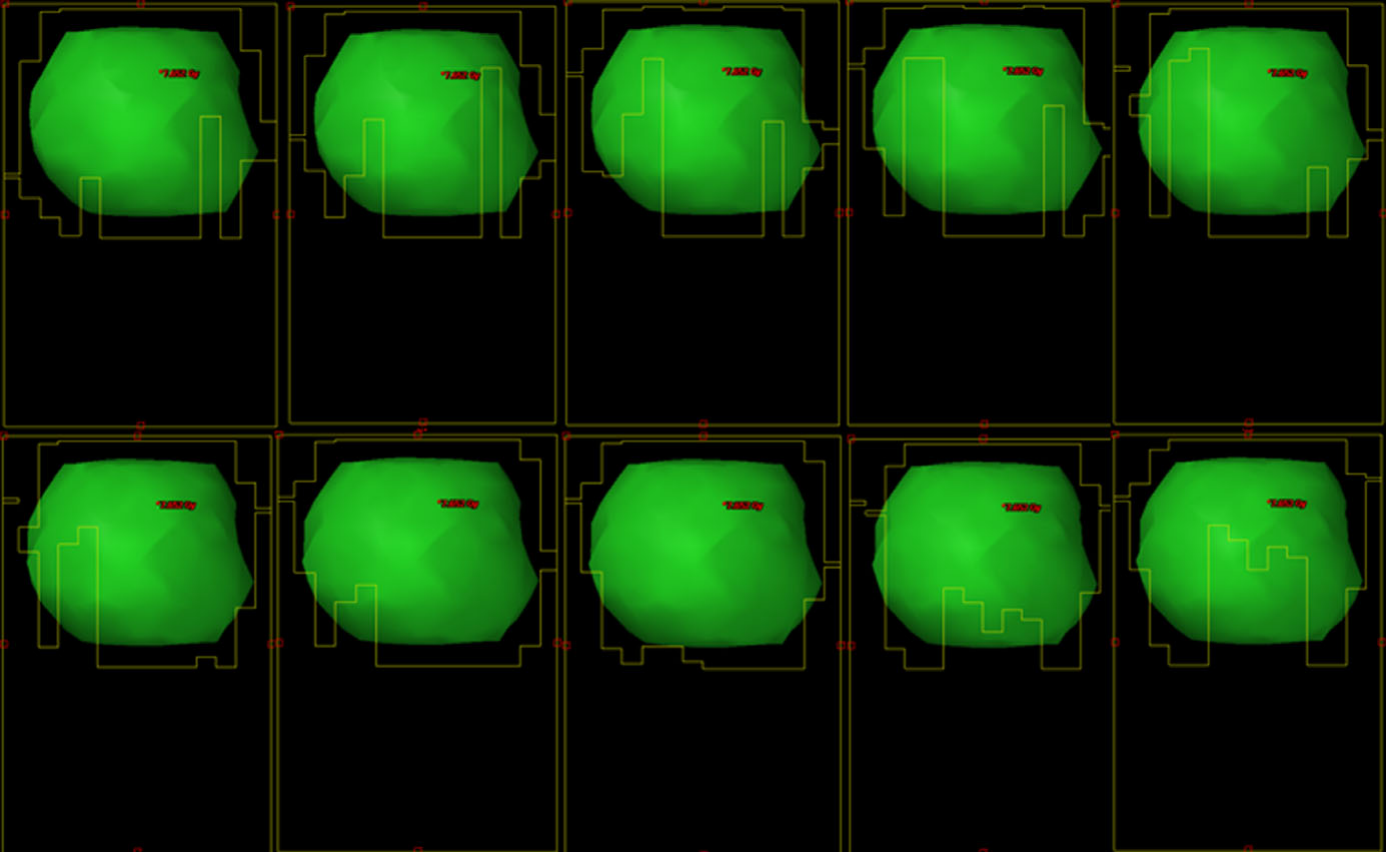
The MLC apertures for ten consecutive control points of a 3D VMAT plan for the lung case #1.

**Figure 3:**
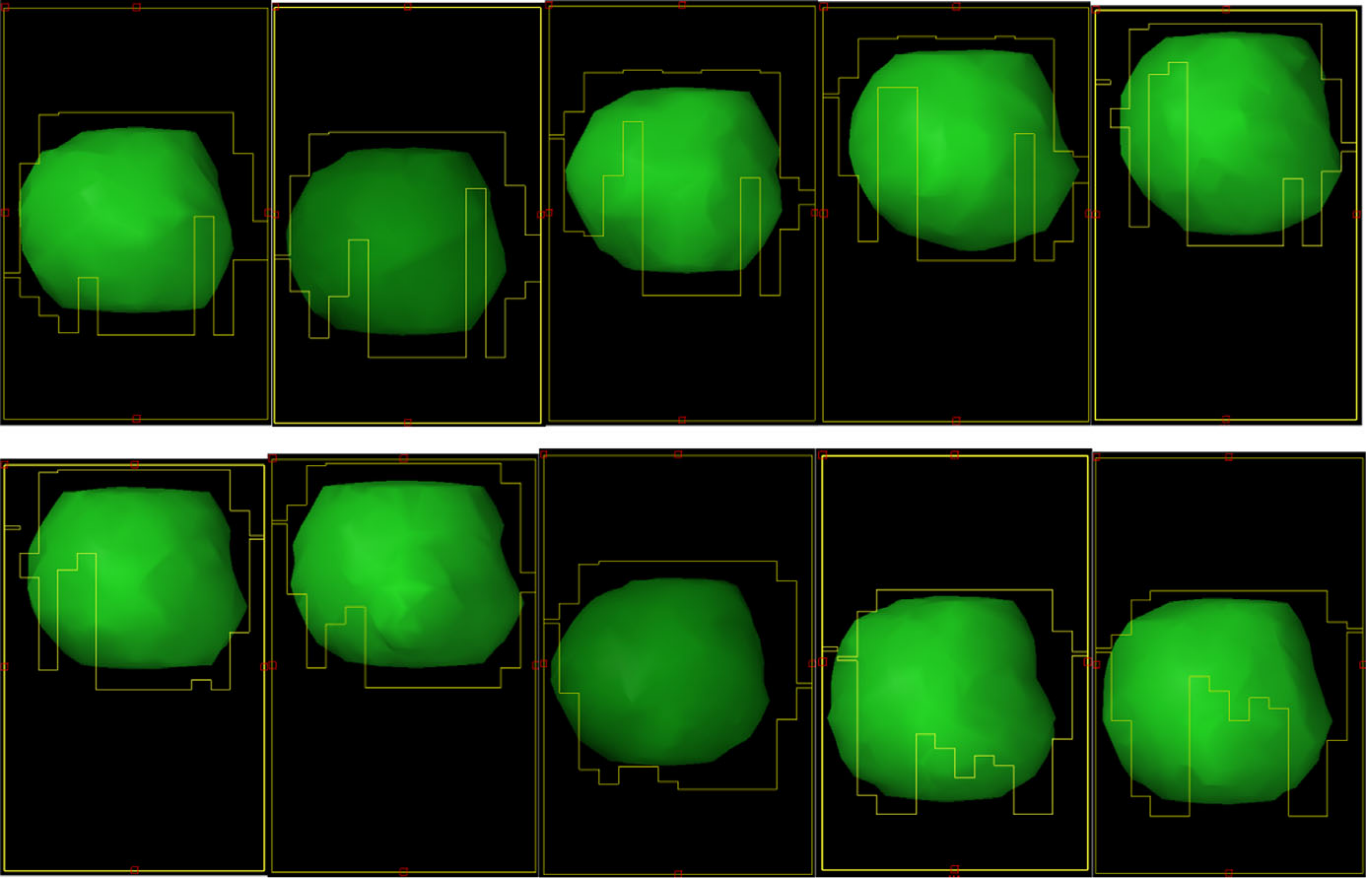
Resultant apertures for ten consecutive control points for the lung case #1. The collimator angle was set at 90° to make sure the MLC can track the tumor motion.

If the projection of the target for the *N*th Phase is shorter than the reference target in the *X*‐direction, or there is no new target under the corresponding (*i*+*k*)th leaf pair, the leaf pair would be closed in the new leaf sequence. On the other hand, if the *i*th leaf pair is originally closed while there is a new target under the corresponding (*i* + *k*)th leaf pair, the (*i* + *k*)th leaf pair should be opened based on its adjacent opened leaf pair and the target projection under these two leaf pairs. Figures 2 and 3 showed the apertures for ten consecutive control points covering a full breathing cycle in the 3D and the 4D VMAT plans. The first picture represents the MLC aperture at 0% phase and the last picture represents the MLC aperture at 90% phase.

### 2.C Dosimetric comparison of 4D VMAT plans with 3D VMAT plans

To investigate the 4D VMAT plan quality, the 4D VMAT plans were compared with their corresponding 3D VMAT plans. It consisted of the following steps (Fig. 1). First, the 4D VMAT plan DICOM file was physically separated into 10 files corresponding to 10 phases based on the known correlation between the target position and the beam aperture of each control point. Second, the 10 sub‐files of the 4D VMAT plan were imported back to the TPS. The dose matrix was calculated on each phase of the 4D CT data set using the corresponding sub‐file of the 4D VMAT plan. Third, the dose matrices from the 10 phases were then deformed to the reference phase to generate a 4D dose matrix summation using the Varian VelocityAI 3.1.0 software. The differences between the deformable and the rigid registration for the QUASAR™ phantom 4D VMAT plans were also studied. The 4D dose matrix summation was imported back to Eclipse to calculate the dose distribution and DVHs for the target and OARs on the reference phase. Fourth, the dosimetric parameters of the 4D plan were compared with those of the ideal 3D VMAT plan using the coverage of planning target volume (PTV) and the sparing of organs‐at‐risk. The conformity indices (CI) were also calculated and compared. The CI was defined as:

$$CI=\frac{TV_{PI}}{PI}\times \frac{TV_{PI}}{TV},$$

Where TV_PI_ is the target volume within the prescribed isodose volume PI, TV is the target volume.

### 2.D 4D VMAT plan verification

**Figure 4:**
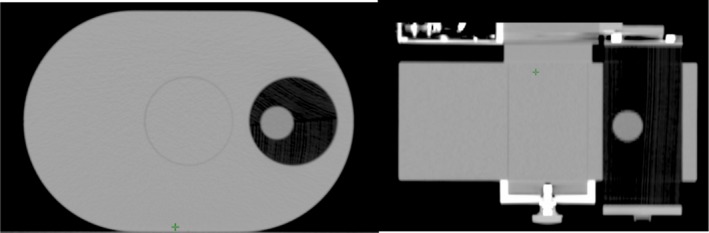
Computed Tomography (CT) images of a QUASAR™ phantom in the transverse plane (left) and coronal plane (right). A 3 cm diameter lung tumor model insert was used for 4D imaging and planning.

3D and 4D plan verifications were performed using EDR 2 film in a QUASAR™ phantom (see Fig. 4). First, the phantom was positioned on the couch using a laser based patient positioning system. Then, the target was accurately localized using kilo‐Voltage (kV) orthogonal setup images to ensure the accuracy of target positioning. 3D VMAT plan was delivered to the static phantom and validated using gamma analysis between the film measurement and the planar dose distribution from the TPS. The gamma index criterion was set to 3%/3 mm. For 4D VMAT plan validation, the QUASAR™ phantom was animated using the real patient‐respiration curve, the amplitude of the respiratory curve of a patient was normalized to match the tumor motion amplitude. The variation in the amplitude and frequency was not translated to change for the internal target. The Varian RPM system was used to synchronize the treatment delivery with the phantom motion. The measured dose distribution was compared with the calculated 4D dose distribution. In our work, the 50% phase of respiratory was used as the beam starting time for the treatment delivery.

**Figure 5:**
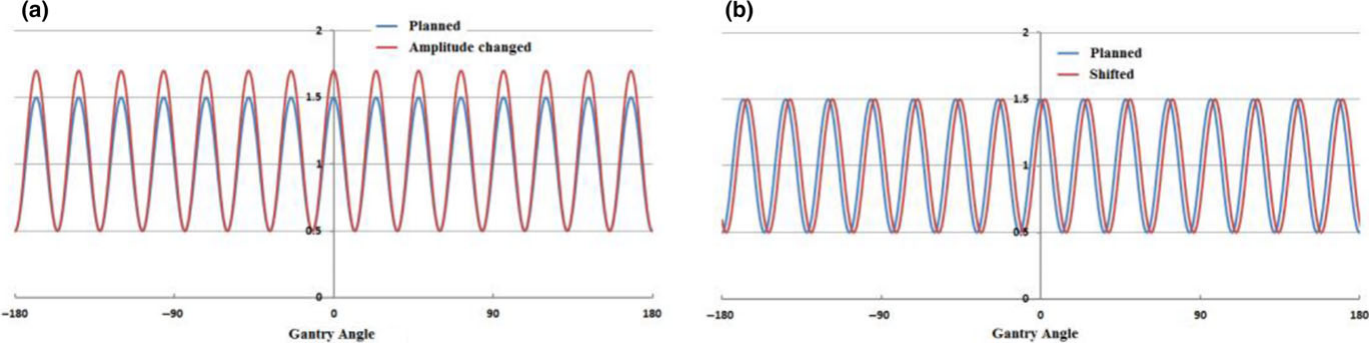
The effects of the breathing amplitude change and phase shift during 4D VMAT deliveries were simulated in Eclipse. The motion amplitude was manually changed by 1 mm, 2 mm, and 3 mm (a) and a 10% breathing cycle shift (b) was introduced during the 4D VMAT deliveries, the resultant dose distributions were calculated and compared with that of the original 4D VMAT plans.

We assumed that the characteristics of the motion are known (from 4D‐CT data) at the treatment planning stage, the adaptive planning strategies from fraction to fraction would not be discussed. However, in this study, the effects of the changes of breathing amplitude and the phase shift between the tumor motion and the treatment delivery to the total dose distribution were simulated using the Eclipse treatment planning system. The motion amplitude was manually changed and the breathing cycle was shifted for the treatment delivery, the resultant dose distributions were calculated and compared with the original 4D VMAT plan dose distributions (see fig. 5).

## 3 Results

### 3.A Dosimetric comparison of 4D VMAT plan with 3D VMAT plan

**Figure 6:**
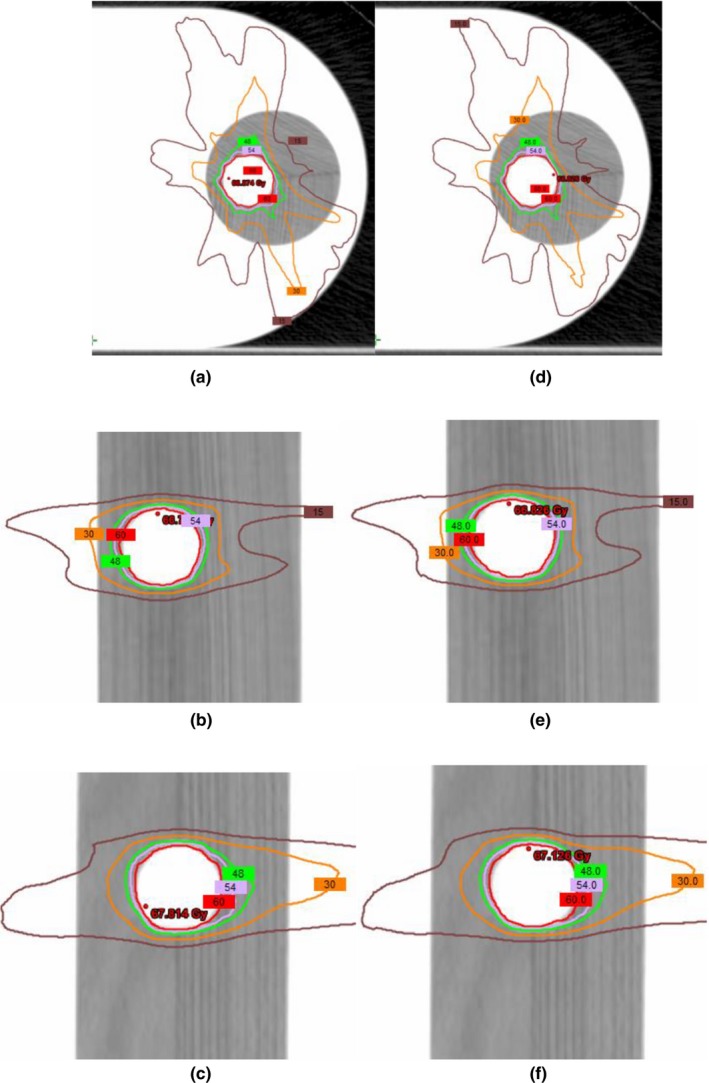
Dose distribution Comparison for 3D (a), (b), (c) and 4D (d), (e), (f) VMAT plans. The 60 Gy, 54 Gy, 48 Gy, 30 Gy, and 15 Gy isodose lines are shown in transversal view (a), (d), coronal view (b), (e) and sagittal view (c), (f). The 4D VMAT plan has comparable dose distribution to that of the 3D plan for the QUASAR™ phantom with periodic motion.

**Figure 7:**
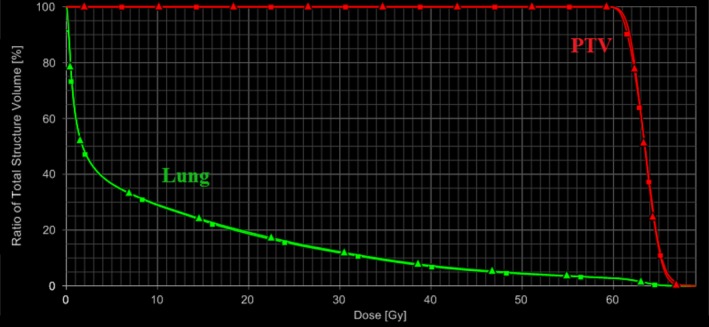
DVH comparison for 3D and 4D VMAT plans. The PTV coverage and the lung DVHs are virtually the same, the PTV maximum dose for the 4D plan decreases from 64.3 to 63.8 Gy for the QUASAR™ phantom with periodic motion.

**Figure 8:**
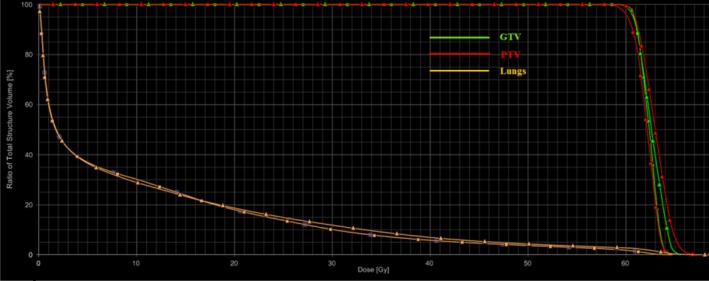
DVH comparison of the 4D VMAT plans calculated with the rigid registration (lines with rectangle symbols) and the deformable registration (lines with triangle symbols). The GTV coverage and the dose to the lungs are similar for both registration methods, though the PTV coverage for the rigid registration is lower (96.5%) than that for the deformable registration (99.5%).

Figure 6 presents the dose distributions of the 3D (a, b, and c) and the 4D (d, e, and f) VMAT plans for the phantom. The 4D VMAT plan quality is comparable to that of the 3D VMAT plan. The DVH comparison in Figure 7 indicates that the PTV coverage is nearly the same for both plans; the maximum dose to the PTV decreases from 64.3 Gy to 63.8 Gy for the 4D VMAT plan. The changes in lung dosimetry are insignificant. Figure 8 compares DVHs of the 4D dose distribution calculated with the rigid registration and the deformable registration. The GTV coverage and the dose to the lungs are similar for both registration methods, though the PTV coverage for the rigid registration is lower (96.5%) than that for the deformable registration (99.5%).

**Figure 9:**
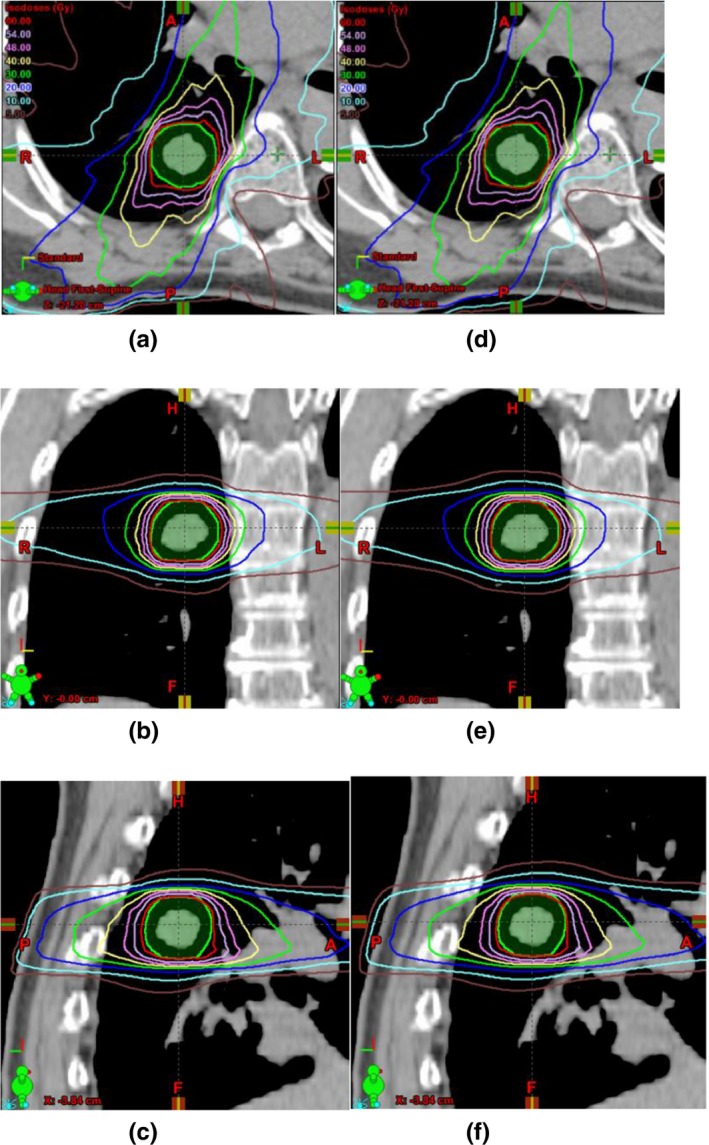
Comparison of isodose distributions for the 3D (a), (b), and (c) and 4D (d), (e), and (f) VMAT plans are shown in the left and right panels respectively. Both plans were generated on the 50% CT image for a lung case with target motion amplitude of 1.6 cm for patient #1.

**Figure 10:**
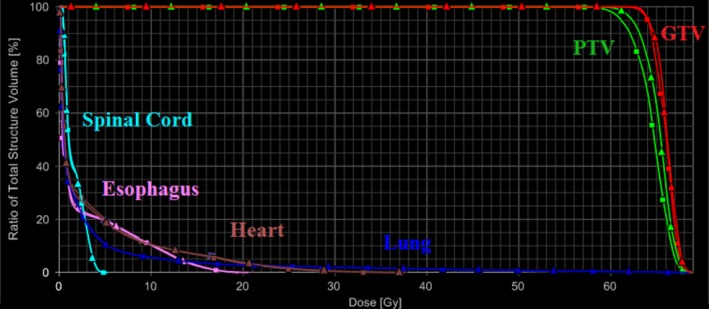
DVH comparison of 4D and 3D VMAT plans for the lung case #1. The lines with triangle symbols represent the 4D VMAT plan whereas the lines with rectangle symbols represent the 3D VMAT plan. The 4D plan has similar GTV coverage and comparable critical structure doses for this patient.

Figures 9 and 10 present the dose distributions and DVHs of 3D and 4D VMAT plans for the patient #1. Comparing the 4D with the 3D plan, the PTV prescription dose coverage decreases from 98.5% to 97.0% (Table 2) while the maximum esophagus dose reduces from 20.7 Gy to 19.6 Gy (Table 3) for the 4D plan.

**Table 2:** Comparison of dose statistics for the GTV and PTV for the patient studies. The 4D VMAT plans have comparable GTV minimum and mean doses to that of the 3D VMAT plans. The PTV coverage decreases from 98.8% to 94.7%, but the mean dose has only 0.8% difference (from 64.3 Gy to 63.8 Gy) and the conformity indices of the PTV for the 4D VMAT plans were comparable to the 3D VMAT plans

Patient no.	GTV				PTV					
	Min dose		Mean dose		Min dose		Coverage (%)		CI	
	3D	4D	3D	4D	3D	4D	3D	4D	3D	4D
1	61.9	62.7	66.1	65.9	57.7	56.1	98.5	97.0	0.80	0.79
2	61.3	61.8	63.7	63.9	57.7	54.1	99.0	92.1	0.86	0.83
3	60.8	60.9	63.3	63.9	58.4	55.5	99.4	93.9	0.77	0.78
4	60.5	60.1	61.8	62.0	54.0	52.3	98.2	97.3	0.81	0.81
5	60.8	60.8	67.8	68.0	52.3	46.1	98.8	93.5	0.72	0.72
Average	61.1	61.3	64.5	64.7	56.0	52.8	98.8	94.7	0.79	0.78

**Table 3:** Dosimetric comparison of mean doses for lung and heart and the maximum doses for spinal cord and esophagus between 3D and 4D VMAT plans. No significant difference is seen between the two sets of plans

Case no.	Lung mean dose (Gy)		Heart mean dose (Gy)		Spinal cord max dose (Gy)		Esophagus max dose (Gy)	
	3D	4D	3D	4D	3D	4D	3D	4D
1	2.8	2.7	3.2	3.3	1.6	1.6	20.7	19.6
2	1.5	1.5	0.7	0.7	5.1	4.9	5.2	4.8
3	1.8	1.8	2.9	2.9	8.5	8.3	10.0	9.7
4	0.9	0.9	3.2	3.1	6.6	6.2	9.9	9.6
5	4.9	4.8	11.2	11.1	10.7	11.7	16.3	15.9
Average	2.4	2.3	4.3	4.2	6.5	6.5	12.4	11.9

Table 2 lists the dosimetric statistics for GTV and PTV for the patient studies. The results show that 100% of the GTV is covered by the prescription dose, the minimum and mean doses to the GTV are nearly invariant; Comparing with the 3D plans, the PTV coverage decreases from 98.8% to 94.7%, and the mean dose drops 0.8% for the 4D plans.

Table 3 lists the dosimetric parameters for various critical structures such as the mean doses for lungs and heart and the maximum doses for spinal cord and esophagus. The average mean lung dose is 2.3 Gy for 4D VMAT plans and 2.4 Gy for 3D VMAT plans. The average mean dose for heart is 4.2 Gy for 4D VMAT plans and 4.3 Gy for 3D VMAT plans. The spinal cord receives an average maximum dose of 6.5 Gy for both the 4D and 3D VMAT plans, and the esophagus average maximum point dose is 11.9 Gy for 4D and 12.4 Gy for 3D VMAT plans, respectively. These data illustrate that there is no significant differences between the 3D and 4D VMAT plans.

### 3.B Plan verification

**Figure 11:**
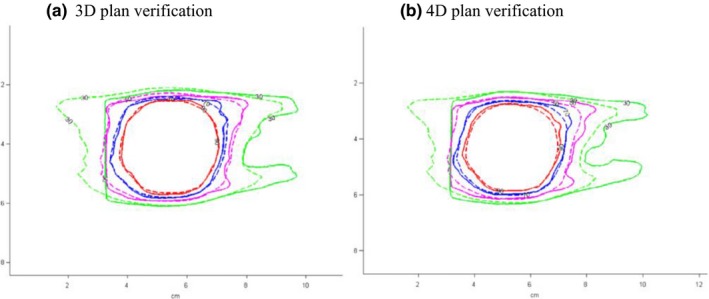
Measured isodose distributions for 3D (a) and 4D (b) VMAT plans. The measured (solid lines) 90%, 70%, 50%, and 30% isodose lines are compared to the calculated isodose lines in the figure. The gamma pass ratio is 98.6% for the 3D VMAT plan and 95.7% for the 4D VMAT plan with the criteria of 3%, 3 mm for the QUASAR™ phantom with periodic motion.

The results of the phantom plan verification for 3D and 4D VMAT plans are shown in Fig. 11. The gamma pass ratio is 98.6% for the 3D VMAT plan and 95.7% for the 4D VMAT plan with the criteria of 3%/ 3 mm.

**Figure 12:**
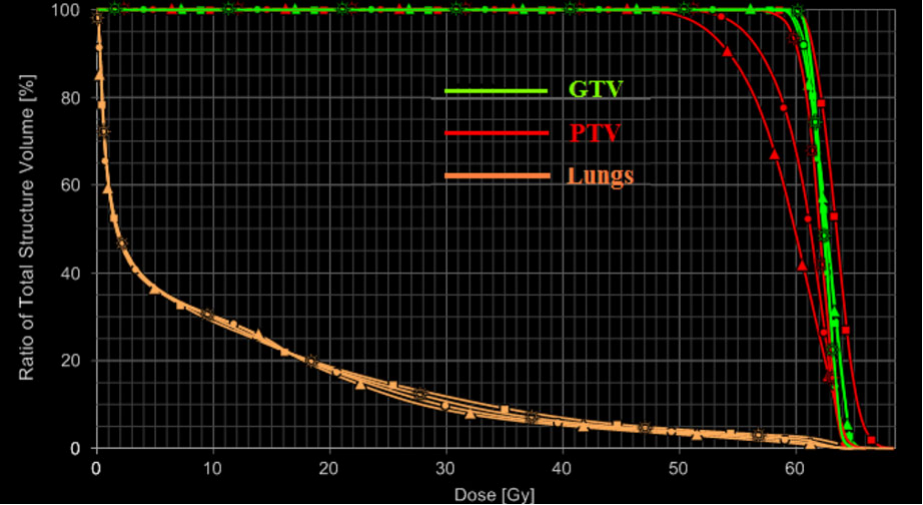
The effects of the breathing amplitude change to the total dose distribution are simulated using the Eclipse treatment planning system. The DVHs for motion amplitude of 1.0 cm (lines with rectangle symbols), 1.1 cm (lines with star symbols), 1.2 cm (lines with dot symbols), and 1.3 cm (lines with triangle symbols) are compared. Dose alterations to GTV and lungs are not significant, but the D95 to the PTV drops from 61.0 Gy to 52.4 Gy when the breathing amplitude changes from 1.0 cm to 1.3 cm during the 4D VMAT plan delivery.

The DVH comparisons for the tumor motion amplitude of 1.0 cm, 1.1 cm, 1.2 cm, and 1.3 cm are shown in Fig. 12. Results indicate that dose alterations to GTV and lungs are not significant, but the D95 to the PTV dropped from 61.0 Gy to 52.4 Gy when the breathing amplitude changed from 1.0 cm to 1.3 cm during the 4D VMAT plan delivery. The DVH comparisons were shown in Fig. 12.

**Figure 13:**
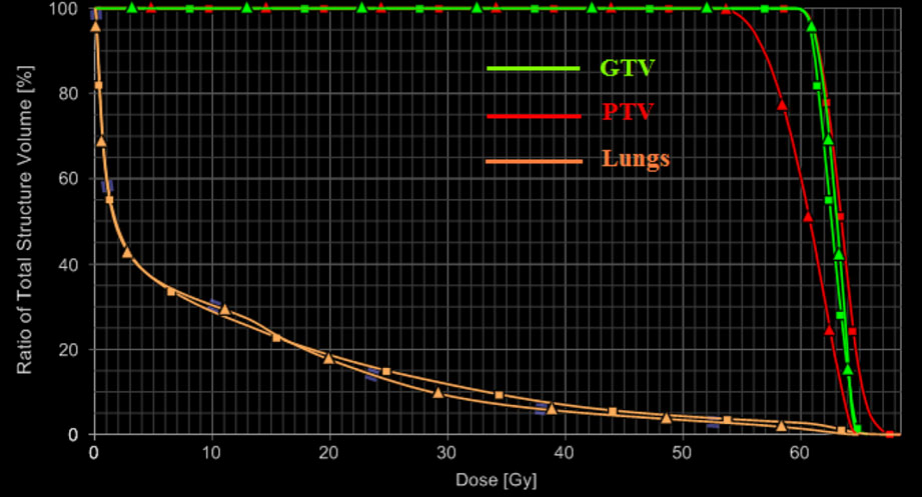
The effect of phase shift during the treatment delivery to the total dose distribution is simulated in Eclipse. The D95 drops from 61.0 Gy to 56.1 Gy when the phase shift between the tumor motion and the treatment delivery is 10%.

Figure 13 shows the effect of phase shift between the tumor motion and the treatment delivery to the total dose distribution simulated in Eclipse. The D95 dropped from 61.0 Gy to 56.1 Gy when the phase shift was 10%. Dose alterations to GTV and lungs were not significant.

**Figure 14:**
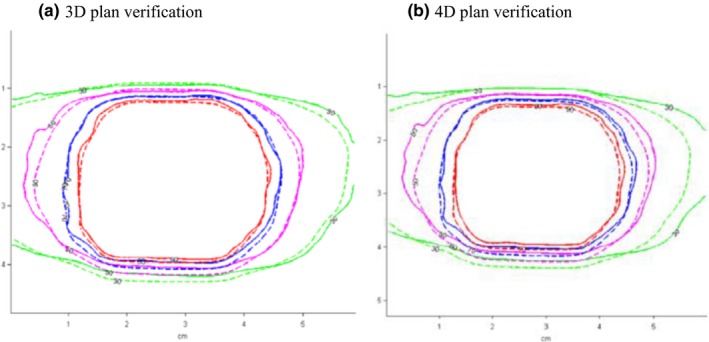
Plan verifications for 3D and 4D VMAT plans. The 30%, 50%, 70%, and 90% isodose lines are shown in solid lines (measured dose distribution) and dashed lines (calculated dose distribution) for patient #1. The gamma pass rate is 94.5% for the 3D VMAT static delivery and 94.1% for the 4D VMAT plan (3%, 3 mm).

**Figure 15:**
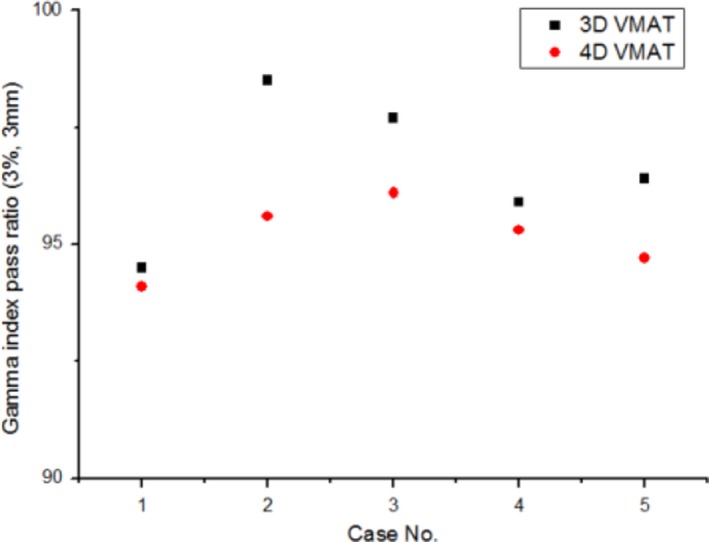
Comparison of 3D and 4D VMAT plan verifications. The average gamma index pass ratio is 96.5% for 3D and 95.2% for 4D VMAT plans, respectively. The gamma pass ratio was more than 90% for all five patients.

The results of the patient plan verification for 3D and 4D VMAT plans are shown in Fig. 14. The measured dose distribution has a good agreement to that of the calculation. The gamma passing ratio is 94.5% and 94.1% for 3D and 4D VMAT plans separately. The statistics of the gamma pass ratio for 3D and 4D VMAT plans is shown in Fig. 15. The average gamma pass ratio is 96.5% for 3D and 95.2% for 4D VMAT plans, respectively.

## 4 Discussion

The 4D VMAT has the potential to improve radiation therapy of periodically moving tumors over 3D VMAT, gating, or tracking methods. Generally, the 4D VMAT plans can be implemented either by independently optimizing each of the phases or by considering all the phases simultaneously.^22^, ^23^, ^24^, ^25^ The inverse planning frameworks proposed by Ma^23^and Chin^24^, ^25^ are realized by incorporating 4D volumetric target and OAR motions directly into the optimization process. During optimization, phase correlated beam apertures are optimized throughout the full range of gantry rotation so that the resulting treatment plans have respiratory phase‐optimized apertures. Our 4D VMAT planning using the DAD method simplifies the optimization process. The plan quality is comparable to an ideal plan. The mean dose is only 0.8% lower than the optimal 3D plan for the PTV and doses to the normal tissues are nearly identical.

The 4D VMAT plan is created based on the patient 4D CT. It is not always true that the 4D CT image set represents the patient motion pattern during treatment delivery, so issues exist with the 4D VMAT plan delivery to a patient. First, the 4D CT scan is usually taken long before the plan delivery. Second, even with 4D CT, the free‐breathing simulation is only a snapshot and a single stochastic sampling of the patient's breathing, thus a change in patient's breathing pattern during the simulation or treatment may greatly affect the dose delivery accuracy. Guckenberger^15^ presented that a single 4D CT scan cannot accurately predict pancreatic tumor motion during delivery for radiosurgery. If 4D cone‐beam CT^18^, ^19^, ^20^is available, the most recent information on the patient's anatomic locations can be used accounting for the tumor motion more effectively and the 4D dose delivery will be more accurate.^26^

The phantom plan maintains the PTV coverage, but for patient plans, the PTV coverage for 4D VMAT plans is lower than 3D VMAT plans. The reasons for the decrease in the PTV coverage are: (a) the reproduction of the breathing motion is essential for the 4D VMAT planning and treatment delivery. For patient plans, audio coaching can reduce variation in the breathing motion, but any uncertainties of the breathing motion will be transferred to the 4D CT images and the treatment planning. (b) The DAD method deforms the optimized MLC apertures to the other phases based on the deformation and translation of target contours, the difference between the DAD MLC aperture deformation and the 3D dose deformation algorithm in Varian VelocityAI introduces uncertainties for the 4D VMAT dose summations, especially when complex deformation and rotation occur in the lung region.

The repeatability of the patients’ breathing pattern may greatly affect the accuracy of the dose delivery. Our experimental measurements show that the gamma pass ratio of the VMAT plans is 95.2%, but we should point out that real patient respiration sometimes exhibits very complicated patterns with continuously changing amplitude and periodicity, drifting baseline and envelope effect.^27^Although several methods discussed in AAPM TG report76,^7^ such as audiovisual biofeedback, breath‐hold, and abdominal compression, can be used to manage the respiratory motion and improve breathing repeatability, the availability of real‐time monitoring of the tumor motion and effective feedback of the tumor motion information play an important role in dealing with realistic clinical situations where breathing irregularities may occur. Action thresholds must be established to determine when a beam interlock must be triggered to account for the amplitude change and phase shift during the treatment delivery. The motion effects should be carefully evaluated and will be the focus of our future work.

## 5 Conclusions

The work presented a 4D VMAT planning technique for dynamic targets using a DAD method. The proposed method is a practical and simple approach to account for both rigid and non‐rigid target motion. The plan quality of the 4D VMAT plans is comparable to the 3D optimal plans in terms of the tumor coverage and the normal tissue sparing. Because the target motion is continuous, this DAD method generates continuous MLC sequences between apertures of successive phases. The 4D VMAT plans were verified with the QUASAR™ phantom, and the effects of the motion amplitude and the phase shift were simulated in Eclipse. The 4D treatment delivery time is the same as the optimal 3D VMAT plan.

## Acknowledgment

The authors would like to thank Edward Brandner for his assistance and comments that greatly improved the manuscript. Dr. Xiang Li and Dr. Tianfang Li were supported in part through the NIH/NCI Cancer Center Support Grant P30 CA008748.

## Conflict of Interest

The authors declare there are no conflicts of interest in connection with this work.

## References

[1] Otto K . Volumetric modulated arc therapy: IMRT in a single gantry arc. Med Phys. 2008;35:310–317. 18293586 10.1118/1.2818738

[2] Verbakel WF , Cuijpers JP , Hoffmans D , Bieker M , Slotman BJ , Senan S . Volumetric intensity‐modulated arc therapy vs. conventionalIMRT in head‐and‐neck cancer: a comparative planning and dosimetricstudy. Int J Radiat Oncol Biol Phys. 2009;74:252–259. 19362244 10.1016/j.ijrobp.2008.12.033

[3] Lagerwaard FJ , Meijer OWM , dervan Hoorn EAP , Verbakel W , Slotman BJ , Senan S . Volumetric modulated arc radiotherapy forvestibularschwannomas. Int J Radiat Oncol Biol Phys. 2009;74:610–615. 19427560 10.1016/j.ijrobp.2008.12.076

[4] Lagerwaard FJ , dervan Hoorn EAP , Verbakel W , Haasbeek CJA , Slotman BJ , Senan S . Whole‐brain radiotherapy with simultaneousintegrated boost to multiple brain metastases using volumetricmodulated arc therapy. Int J Radiat Oncol Biol Phys. 2009;75:253–259. 19577856 10.1016/j.ijrobp.2009.03.029

[5] Cozzi L , Dinshaw KA , Shrivastava SK , et al. A treatment planning study comparing volumetric arcmodulation with RapidArc and fixed field IMRT for cervix uteri radiotherapy. Radiother Oncol. 2008;89:180–191. 18692929 10.1016/j.radonc.2008.06.013

[6] Verbakel WFAR , Senan S , Cuijpers JP , Slotman BJ , Lagerwaard FJ . Rapid delivery of stereotactic radiotherapy for peripherallung tumors using volumetric intensity‐modulated arcs. Radiother Oncol. 2009;93:122–124. 19552979 10.1016/j.radonc.2009.05.020

[7] Keall PJ , Mageras GS , Balter JM , et al. Themanagement of respiratory motion in radiation oncology report of AAPMTask Group 76. Med Phys. 2006;33:3874–3900. 17089851 10.1118/1.2349696

[8] Stevens CW , Munden RF , Forster KM , et al. Respiratory‐driven lung tumormotion is independent of tumor size, tumor location, and pulmonary function. Int J Radiat Oncol Biol Phys. 2001;51:62–68. 11516852 10.1016/s0360-3016(01)01621-2

[9] Benedict SH , Yenice KM , Followill D , et al. Stereotactic body radiation therapy: the reportof AAPM Task Group 101. Med Phys. 2010;37:4078–4101. 20879569 10.1118/1.3438081

[10] Coolens C , Webb S , Shirato H , Nishioka K , Evans PM . A margin model to account for respiration‐induced tumour motion and its variability. Phys Med Biol. 2008;53:4317–4330. 18653921 10.1088/0031-9155/53/16/007

[11] Shirato H , Shimizu S , Kunieda T , et al. Physical aspects of a real‐time tumor‐tracking systemfor gated radiotherapy. Int J Radiat Oncol Biol Phys. 2000;48:1187–1195. 11072178 10.1016/s0360-3016(00)00748-3

[12] Vedam SS , Keall PJ , Kini VR , Mohan R . Determining parametersfor respiration‐gated radiotherapy. Med Phys. 2001;28:2139–2146. 11695776 10.1118/1.1406524

[13] Nicolini G , Vanetti E. , Clivio EA , Fogliata A , Cozzi L . Pre‐clinical evaluation of respiratory‐gated delivery of volumetric modulated arc therapy with RapidArc. Phys Med Biol. 2010;55:N347–N357. 20484779 10.1088/0031-9155/55/12/N01

[14] Qian J , Xing L , Liu W , Luxton G . Dose verification for respiratory‐gated volumetric modulated arc therapy. Phys Med Biol. 2011;56:4827–4838. 21753232 10.1088/0031-9155/56/15/013 PMC3360016

[15] Sawant A , Venkat R , Srivastava V , et al. Management of three‐dimensional intrafractionalmotionthrough real‐time DMLC tracking. Med Phys. 2008;35:2050–2061. 18561681 10.1118/1.2905355 PMC2809733

[16] Gui M , Feng Y , Yi B , Dhople AA , Yu C . Four‐dimensional intensity modulated radiation therapy planning for dynamic tracking using a direct aperture deformation (DAD) method. Med Phys. 2010;37:1966–1975. 20527530 10.1118/1.3319498 PMC2862055

[17] Sun B , Rangaraj D , Papiez L , Oddiraju S , Yang D , Li HH . Target tracking using DMLC for volumetric modulated arc therapy: a simulation study. Med Phys. 2010;37:6116–6124. 21302768 10.1118/1.3511516 PMC2997810

[18] Ma Y , Chang D , Keall P , et al. Inverse planning for four‐dimensional (4D) volumetric modulated arc therapy. Med Phys. 2010;37:5627–5633. 10.1118/1.3497271 PMC2967715 21158274

[19] Chin E , Otto K . Investigation of a novel algorithm for true 4D‐VMAT planning with comparison to tracked, gated and static delivery. Med Phys. 2011;38:2698–2707. 21776806 10.1118/1.3578608

[20] Chin E , Loewen SK , Nichol A , Otto K . 4D VMAT, gated VMAT, and 3D VMAT for stereotactic body radiation therapy in lung. Phys Med Biol. 2013;58:749–770. 23324560 10.1088/0031-9155/58/4/749

[21] Guckenberger M , Wilbert J , Meyer J , Baier K , Richter A , Flentje M . Is a single respiratory correlated 4D CT study sufficient for evaluation of breathing motion? Int J Radiat Oncol Biol Phys. 2007;67:1352–1359. 17394941 10.1016/j.ijrobp.2006.11.025

[22] Vedam S , Dong L , Zhang J , et al. Impact of respiration‐induced tumor motion uncertainties on adaptive treatment delivery strategies: a comparison through repeat 4D CT imaging. Int J Radiat Oncol Biol Phys. 2006;66:614.

[23] Britton KR , Starkschall G , Pan T , Chang J , Mohan R , Komaki R . Time trends in mobility and size of target volumes for locally advanced stage III non‐small‐cell lung cancer patients using serial our‐dimensional computed tomography (4‐D CT). Int J Radiat Oncol Biol Phys. 2006;66:468.

[24] Sonke J , Zijp L , Remeijer P , Van Herk M . Respiratory correlated cone beam CT. Med Phys. 2005;32:1176–1186. 15895601 10.1118/1.1869074

[25] Li T , Schreibmann E , Yang Y , Xing L . Motion correction for improved target localization with on‐board cone‐beam computed tomography. Phys Med Biol. 2006;51:253–267. 16394337 10.1088/0031-9155/51/2/005

[26] Li T , Xing L , Munro P , et al. Four‐dimensional cone‐beam computed tomography using an on‐board imager. Med Phys. 2006;33:3825–3833. 17089847 10.1118/1.2349692

[27] Fu W , Yang Y , Yue NJ , Heron DE , Huq MS . A cone beam CT‐guided online plan modification technique to correct interfractional anatomic changes for prostate cancer IMRT treatment. Phys Med Biol. 2009;54:1691–1703. 19242051 10.1088/0031-9155/54/6/019

